# MRI Utilisation in Acute Lateral Ankle Ligament Injuries: A Clinical Audit of Adherence to Evidence-Based Imaging Guidelines and Impact on Management

**DOI:** 10.7759/cureus.117537

**Published:** 2026-10-06

**Authors:** Nay Aung Zin, Azwar Anjum, Wasiq Bin Tariq, Khadheeja Abdulla, Reham Bukhari, Wii Ra Aung, Min Maung Maung

**Affiliations:** 1 Department of Orthopaedics and Traumatology, Kulhudhuffushi Regional Hospital, Kulhudhuffushi, MDV; 2 Department of General Surgery, Kulhudhuffushi Regional Hospital, Kulhudhuffushi, MDV; 3 Department of Emergency Medicine, Kulhudhuffushi Regional Hospital, Kulhudhuffushi, MDV; 4 Department of Pediatrics, Kulhudhuffushi Regional Hospital, Kulhudhuffushi, MDV; 5 Department of Orthopaedics, University of Medicine, Magway, Magway, MMR; 6 Department of Orthopaedics and Traumatology, Central Clinic, Malé, MDV

**Keywords:** ankle sprain, anterior talofibular ligament, clinical audit, lateral ankle ligament injury, magnetic resonance imaging, mri utilisation, quality improvement

## Abstract

Background

Acute lateral ankle ligament injuries are common musculoskeletal presentations and are usually diagnosed through clinical assessment supported by plain radiography when indicated. Magnetic resonance imaging (MRI) provides detailed assessment of ligamentous, osteochondral, tendinous, and occult osseous pathology but is generally recommended selectively rather than routinely. Inappropriate or poorly justified MRI utilisation may increase resource use without altering clinical management.

Objective

To evaluate MRI utilisation in patients with acute lateral ankle ligament injuries at Kulhudhuffushi Regional Hospital, assess adherence to predefined evidence-based imaging criteria, identify deficiencies in clinical documentation, and determine whether appropriately indicated MRI was associated with changes in patient management.

Methods

A retrospective clinical audit was conducted at Kulhudhuffushi Regional Hospital, Maldives. Records of patients managed between February 1, 2025 and May 31, 2026 were reviewed. Fifty-five patients aged 14-65 years who presented within one to three weeks of acute traumatic ankle injury and underwent MRI for suspected lateral ankle ligament injury were included. Clinical documentation, plain radiographs, MRI reports, indications for MRI, and subsequent management were evaluated against predefined standards derived from international recommendations. MRI requests were classified as meeting evidence-based criteria when at least one recognised indication was documented before imaging; requests without a documented recognised indication were classified as potentially inappropriate. The association between MRI appropriateness and subsequent management change was evaluated using Fisher's exact test, with ORs and 95% CIs.

Results

All 55 patients (100%) underwent plain radiography before MRI. Forty-two MRI requests (76.4%) met predefined evidence-based indications, below the audit target of ≥90%, while 13 (23.6%) had no documented recognised indication and were classified as potentially inappropriate. Documentation was suboptimal for weight-bearing status (43, 78.2%), ligament-specific examination (37, 67.3%), syndesmotic testing (22, 40.0%), and the Ottawa Ankle Rules (16, 29.1%).

Anterior talofibular ligament (ATFL) injury was the most frequent MRI finding, occurring in 32 patients (58.2%). Other findings included bone bruising in 11 (20.0%), combined ATFL and calcaneofibular ligament (CFL) injury in nine (16.4%), syndesmotic injury in six (10.9%), osteochondral lesions in four (7.3%), occult fractures in three (5.5%), and tendon injuries in three (5.5%). MRI resulted in a change in management in 19 patients (34.5%). Management changed in 18 of 42 patients (42.9%) whose MRI met evidence-based indications compared with one of 13 patients (7.7%) whose MRI lacked a documented recognised indication (OR: 9.00, 95% CI: 1.07-75.70; Fisher's exact test, p = 0.022).

Conclusions

MRI was most clinically useful when requested for patients with documented evidence-based indications. Although compliance with first-line plain radiography was complete, deficiencies were identified in clinical documentation and MRI selection. Appropriately indicated MRI was associated with a significantly higher likelihood of management change. A structured ankle assessment proforma, standardised MRI request criteria, improved rehabilitation pathways, and clinician education are proposed, followed by a re-audit to assess improvement.

## Introduction

Acute ankle trauma is a common presentation in emergency departments, primary care settings, and orthopaedic clinics. Although most ankle injuries can initially be managed without advanced imaging, appropriate investigation is essential to exclude fractures and identify clinically significant associated injuries. Current imaging recommendations support plain radiography as the initial investigation when fracture is clinically suspected, while magnetic resonance imaging (MRI) is generally reserved for selected patients with persistent symptoms, negative or inconclusive radiographs, or suspected occult fracture, syndesmotic injury, osteochondral lesion, tendon injury, or significant ligament disruption [1,2].

Ankle sprains account for a substantial proportion of musculoskeletal and sports-related injuries, with lateral ankle sprains representing the most common pattern [3]. The lateral ligament complex comprises the anterior talofibular ligament (ATFL), calcaneofibular ligament (CFL), and posterior talofibular ligament (PTFL). The ATFL is particularly vulnerable during plantarflexion and inversion. Although most patients recover with appropriate functional treatment and rehabilitation, inadequate assessment or management may contribute to persistent pain, recurrent sprains, impaired function, and chronic ankle instability [4-6].

Contemporary clinical guidelines emphasise that acute lateral ankle sprains should primarily be evaluated through a structured history and physical examination [5-7]. Assessment should include the mechanism and timing of injury, ability to bear weight, localisation of tenderness, swelling and bruising, range of movement, neurovascular status, and ligament-specific stability testing. The anterior drawer and talar tilt tests may assist in evaluating the ATFL and CFL, while squeeze and external rotation stress tests may raise suspicion of syndesmotic injury [5-7].

The Ottawa Ankle Rules, developed and validated by Stiell and colleagues, provide a validated clinical decision tool for determining whether radiography is required following acute ankle or midfoot trauma [8]. A systematic review demonstrated that these rules have high sensitivity for excluding clinically important fractures when correctly applied [9]. Plain radiographs, however, have limited ability to demonstrate ligament tears, cartilage injuries, bone marrow abnormalities, tendon pathology, and some nondisplaced or occult fractures.

MRI provides detailed assessment of the lateral ligament complex, syndesmosis, tendons, articular cartilage, and bone marrow. Nevertheless, routine MRI is not recommended for uncomplicated acute ankle sprains because most patients can initially be managed according to clinical findings and plain radiography [1,2,4,5]. Recent evidence supports MRI for characterising acute ligament pathology, while ultrasonography may also provide useful assessment in appropriately selected patients [10-12]. Advanced imaging is therefore most appropriately considered when symptoms persist despite initial treatment, when the diagnosis remains uncertain, or when a high-grade ligament injury, syndesmotic disruption, osteochondral lesion, tendon injury, occult fracture, or mechanical instability is suspected. National guidance similarly supports clinical assessment and plain radiography before escalation to advanced imaging [13].

Unselective MRI requesting may increase healthcare expenditure, place additional demand on radiology services, prolong waiting times, and identify abnormalities that do not alter management. Conversely, appropriately selected MRI may reveal clinically relevant associated injuries and influence immobilisation, weight-bearing recommendations, rehabilitation, further investigation, specialist referral, or surgical planning.

This clinical audit therefore aimed to evaluate MRI utilisation in patients with acute lateral ankle ligament injuries at Kulhudhuffushi Regional Hospital, assess adherence to predefined evidence-based imaging recommendations, evaluate the quality of clinical documentation, and determine whether appropriately indicated MRI was associated with subsequent changes in patient management.

## Materials and methods

Study design and setting

This retrospective clinical audit was conducted in the Department of Orthopaedics and Traumatology at Kulhudhuffushi Regional Hospital, Maldives. Authors with other institutional affiliations collaborated on the analysis and manuscript; those affiliations do not indicate that patients were recruited from other institutions. The audit evaluated the utilisation of MRI in patients with suspected acute lateral ankle ligament injuries against predefined evidence-based imaging standards. Medical records from February 1, 2025, to May 31, 2026, were reviewed, and data collection and analysis were performed in June 2026.

Study population

Patients presenting with suspected acute lateral ankle ligament injury during the audit period were identified from the hospital's electronic medical records and radiology information system. Of 78 patients screened, 55 (70.5%) fulfilled the predefined eligibility criteria and were included in the final MRI cohort.

Patients were eligible if they were aged 14-65 years, had an acute traumatic ankle injury with clinical suspicion of lateral ligament injury, presented within one to three weeks of injury, underwent plain ankle radiography before MRI, underwent MRI during the same episode of care, and had complete clinical records and MRI reports available for review.

Patients were excluded if they had a radiographically confirmed fracture requiring definitive fracture management, an open ankle injury, previous ankle ligament reconstruction or major ankle surgery, chronic ankle instability or symptoms lasting more than six weeks, inflammatory arthropathy, infection, neoplastic disease, pathological fracture, incomplete clinical records, or an unavailable MRI report.

Audit standards and MRI appropriateness criteria

Audit standards were developed from recommendations of the American College of Radiology (ACR) Appropriateness Criteria, evidence-based ankle sprain guidelines, the Journal of Orthopaedic & Sports Physical Therapy clinical practice guideline, the ROAST consensus statement, the Ottawa Ankle Rules, and National Institute for Health and Care Excellence guidance [1,2,4-9,13].

The following percentage targets were predefined locally for this audit: documentation of the mechanism of injury (≥95%), weight-bearing status (≥95%), ligament-specific examination (≥90%), plain radiography before MRI (100%), MRI meeting predefined evidence-based indications (≥90%), initial conservative management (≥95%), and a documented post-MRI management plan (≥95%).

An MRI request was considered to meet predefined evidence-based criteria when at least one recognised indication was documented before imaging. These included persistent pain or swelling despite appropriate initial conservative management; difficulty or inability to bear weight despite negative or inconclusive radiographs; suspected syndesmotic injury; suspected high-grade or complete lateral ligament injury; objective ankle instability; suspected osteochondral lesion of the talus; suspected occult fracture; suspected tendon injury; mechanical symptoms such as locking, catching, or recurrent giving way; or diagnostic uncertainty in which MRI was expected to alter management [1,2,4,5,10-12].

Requests without any documented recognised indication were classified as potentially inappropriate based on the available clinical documentation. This terminology was used because the retrospective design could not exclude the possibility that a clinically appropriate indication had been present but was not documented.

Data collection

Data were retrospectively extracted from outpatient and emergency department records, electronic medical records, plain radiographs, MRI reports, and treatment documentation using a standardised audit data collection form. Data extraction was performed by the principal investigator, and uncertain cases regarding MRI appropriateness or clinical documentation were reviewed by the supervising consultant.

Variables included patient demographics, side and mechanism of injury, time from injury to presentation, weight-bearing status, clinical examination findings, ligament-specific and syndesmotic testing, Ottawa Ankle Rule documentation, plain radiographic findings, documented MRI indication, MRI findings, initial conservative management, post-MRI management, correlation between the initial clinical diagnosis and MRI findings, and whether MRI resulted in a change in management.

Outcome measures

The primary outcome was the proportion of MRI examinations meeting predefined evidence-based indications. Secondary outcomes included compliance with individual audit standards, the frequency and spectrum of MRI abnormalities, agreement between the initial clinical diagnosis and MRI findings, the proportion of patients whose management changed following MRI, and deficiencies in clinical documentation and MRI utilisation.

Statistical analysis

Data were entered into Microsoft Excel (Microsoft Corporation, Redmond, WA, USA) and analysed using IBM SPSS Statistics for Windows, Version 27.0 (IBM Corp., Armonk, NY, USA). Continuous variables were summarised as mean ± standard deviation (SD), while categorical variables were reported as frequencies and percentages. Compliance with each audit standard was calculated as the proportion of patients meeting the predefined target.

The association between MRI appropriateness and subsequent change in management was assessed using Fisher's exact test because of small expected cell counts. Odds ratios (ORs) with 95% confidence intervals (CIs) were calculated to estimate the strength of association. A two-sided p-value of <0.05 was considered statistically significant.

Ethical considerations

This project was conducted as a clinical audit/quality improvement activity using routinely collected clinical data at Kulhudhuffushi Regional Hospital. Patient identifiers were removed from the audit dataset before analysis. No names, identification numbers, or other patient-identifying details are included in this manuscript. The project was conducted within the hospital's clinical governance framework (clinical audit registration/reference number: KRH/QID/AUD/2026/008). As this project constituted clinical audit rather than research, formal Institutional Review Board (IRB)/research ethics committee review and individual participant consent were not required in accordance with institutional requirements.

## Results

Patient characteristics

A total of 78 patients (100%) were screened for eligibility. After application of the predefined inclusion and exclusion criteria, 55 (70.5%) were included in the final analysis, and 23 (29.5%) were excluded (Figure 1). Included patients underwent MRI within one to three weeks of injury.

**Figure 1:**
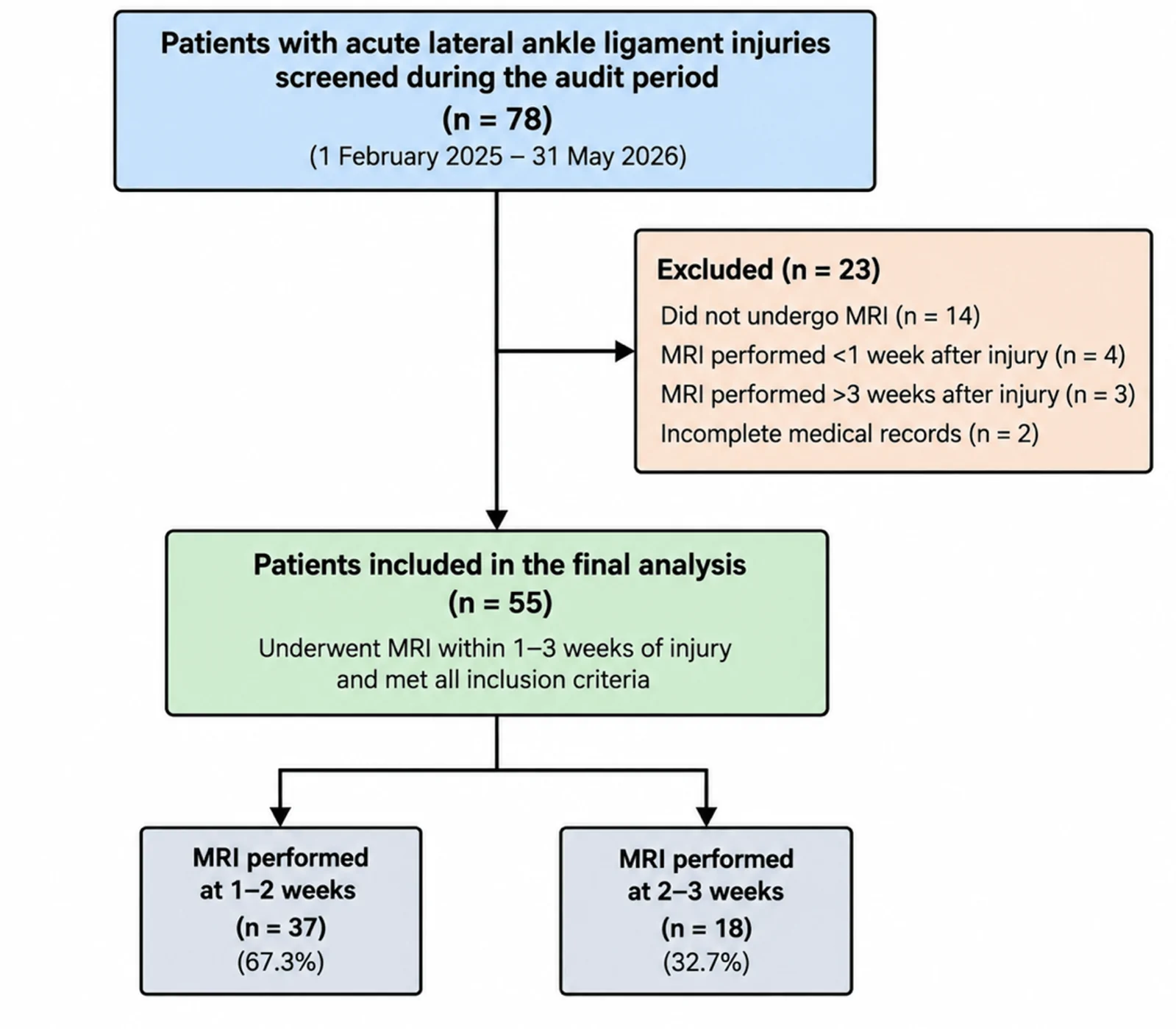
Flow diagram illustrating patient screening and inclusion in the clinical audit. A total of 78 patients presenting with acute lateral ankle ligament injuries between February 1, 2025, and May 31, 2026, were screened for eligibility. Of the 78 patients screened, 55 (70.5%) were included, and 23 (29.5%) were excluded after application of the eligibility criteria. Abbreviation: MRI, magnetic resonance imaging.

The mean age was 32.8 ± 12.4 years (range: 14-65 years). Of the 55 patients, 34 (61.8%) were male, and 21 (38.2%) were female. The right ankle was affected in 30 patients (54.5%) and the left ankle in 25 (45.5%). The most common mechanism was sports-related trauma (21, 38.2%), followed by twisting while walking (17, 30.9%), missed steps or stairs (9, 16.4%), road traffic accidents (5, 9.1%), and other mechanisms (3, 5.5%). Thirty-seven patients (67.3%) presented between one and two weeks after injury, while 18 (32.7%) presented between two and three weeks. Baseline demographic and injury characteristics are summarised in Table 1.

**Table 1:** Baseline demographic and injury characteristics of patients included in the clinical audit (n = 55). The mean age of the study population was 32.8 ± 12.4 years, with an age range of 14-65 years. Data are presented as numbers and percentages unless otherwise stated.

Characteristic	n (%)
Sex	
Male	34 (61.8)
Female	21 (38.2)
Side of injury	
Right	30 (54.5)
Left	25 (45.5)
Mechanism of injury	
Sports-related injury	21 (38.2)
Twisting injury while walking	17 (30.9)
Missed step/stairs	9 (16.4)
Road traffic accident	5 (9.1)
Other	3 (5.5)
Time from injury to presentation	
1-2 weeks	37 (67.3)
2-3 weeks	18 (32.7)

Clinical assessment and documentation

Documentation of the mechanism of injury was present in 53 patients (96.4%), swelling in 51 (92.7%), site of tenderness in 48 (87.3%), neurovascular status in 46 (83.6%), and weight-bearing status in 43 (78.2%). Ligament-specific examination was documented in 37 patients (67.3%). The anterior drawer test was documented in 31 (56.4%), talar tilt test in 24 (43.6%), and syndesmotic tests in 22 (40.0%). The Ottawa Ankle Rules were documented in 16 patients (29.1%). Clinical assessment documentation is summarised in Table 2.

**Table 2:** Documentation of clinical assessment in patients with acute lateral ankle ligament injuries (n = 55). Values are presented as the number and percentage of patients in whom each clinical assessment parameter was documented in the medical record.

Clinical assessment parameter	Documented, n (%)
Mechanism of injury	53 (96.4)
Swelling	51 (92.7)
Site of tenderness	48 (87.3)
Neurovascular examination	46 (83.6)
Weight-bearing status	43 (78.2)
Ligament-specific examination	37 (67.3)
Anterior drawer test	31 (56.4)
Talar tilt test	24 (43.6)
Syndesmotic tests	22 (40.0)
Ottawa Ankle Rules	16 (29.1)

Initial radiographic assessment

All 55 patients (100%) underwent plain ankle radiography before MRI. No fracture was identified in 49 patients (89.1%), four (7.3%) demonstrated soft-tissue swelling, and two (3.6%) had small avulsion fractures that were managed conservatively.

MRI indications and appropriateness

Persistent pain and/or swelling was the most frequently documented indication for MRI, occurring in 29 patients (52.7%). Other indications included suspected high-grade ligament injury in 18 (32.7%), clinical instability in 13 (23.6%), suspected syndesmotic injury in eight (14.5%), difficulty with weight-bearing in seven (12.7%), suspected osteochondral lesion in five (9.1%), suspected occult fracture in four (7.3%), and suspected tendon injury in three (5.5%). More than one indication could be documented for an individual patient. Thirteen MRI requests (23.6%) had no documented recognised indication.

Overall, 42 of 55 MRI examinations (76.4%) met the predefined evidence-based criteria, while 13 (23.6%) were classified as potentially inappropriate based on the available clinical documentation. The predefined audit target of ≥90% appropriate MRI utilisation was therefore not achieved (Table 3).

**Table 3:** Documented clinical indications and appropriateness of MRI requests in patients with acute lateral ankle ligament injuries (n = 55). More than one clinical indication could be documented for an individual patient; therefore, percentages for MRI indications do not total 100%. MRI examinations were classified as meeting predefined evidence-based criteria when at least one recognised indication was documented before imaging. Requests without a documented recognised indication were classified as potentially inappropriate based on the available clinical documentation. Abbreviation: MRI, magnetic resonance imaging.

MRI indication	n (%)
Persistent pain and/or swelling	29 (52.7)
Suspected high-grade ligament injury	18 (32.7)
Clinical instability	13 (23.6)
Suspected syndesmotic injury	8 (14.5)
Difficulty with weight-bearing	7 (12.7)
Suspected osteochondral lesion	5 (9.1)
Suspected occult fracture	4 (7.3)
Suspected tendon injury	3 (5.5)
No documented recognised indication	13 (23.6)
MRI appropriateness	
Met predefined evidence-based criteria	42 (76.4)
Potentially inappropriate based on available documentation	13 (23.6)

MRI findings

ATFL injury was the most common MRI finding, occurring in 32 patients (58.2%). Bone bruising was identified in 11 patients (20.0%), combined ATFL and CFL injury in nine (16.4%), and syndesmotic injury in six (10.9%). Osteochondral lesions were identified in four patients (7.3%), occult fractures in three (5.5%), and tendon injuries in three (5.5%). Seven MRI examinations (12.7%) demonstrated no significant structural abnormality. Some patients had more than one MRI finding; therefore, the categories were not mutually exclusive (Table 4 and Figure 2). Representative radiographic and MRI appearances from a patient in the audit cohort are shown in Figure 3.

**Table 4:** MRI findings in patients with acute lateral ankle ligament injuries (n = 55). Some patients had more than one MRI finding; therefore, the categories are not mutually exclusive, and the percentages do not total 100%. Abbreviations: MRI, magnetic resonance imaging; ATFL, anterior talofibular ligament; CFL, calcaneofibular ligament.

MRI finding	n (%)
Anterior talofibular ligament (ATFL) injury	32 (58.2)
Bone bruising	11 (20.0)
Combined ATFL and calcaneofibular ligament (CFL) injury	9 (16.4)
Syndesmotic injury	6 (10.9)
Osteochondral lesion	4 (7.3)
Occult fracture	3 (5.5)
Tendon injury	3 (5.5)
No significant abnormality	7 (12.7)

**Figure 2:**
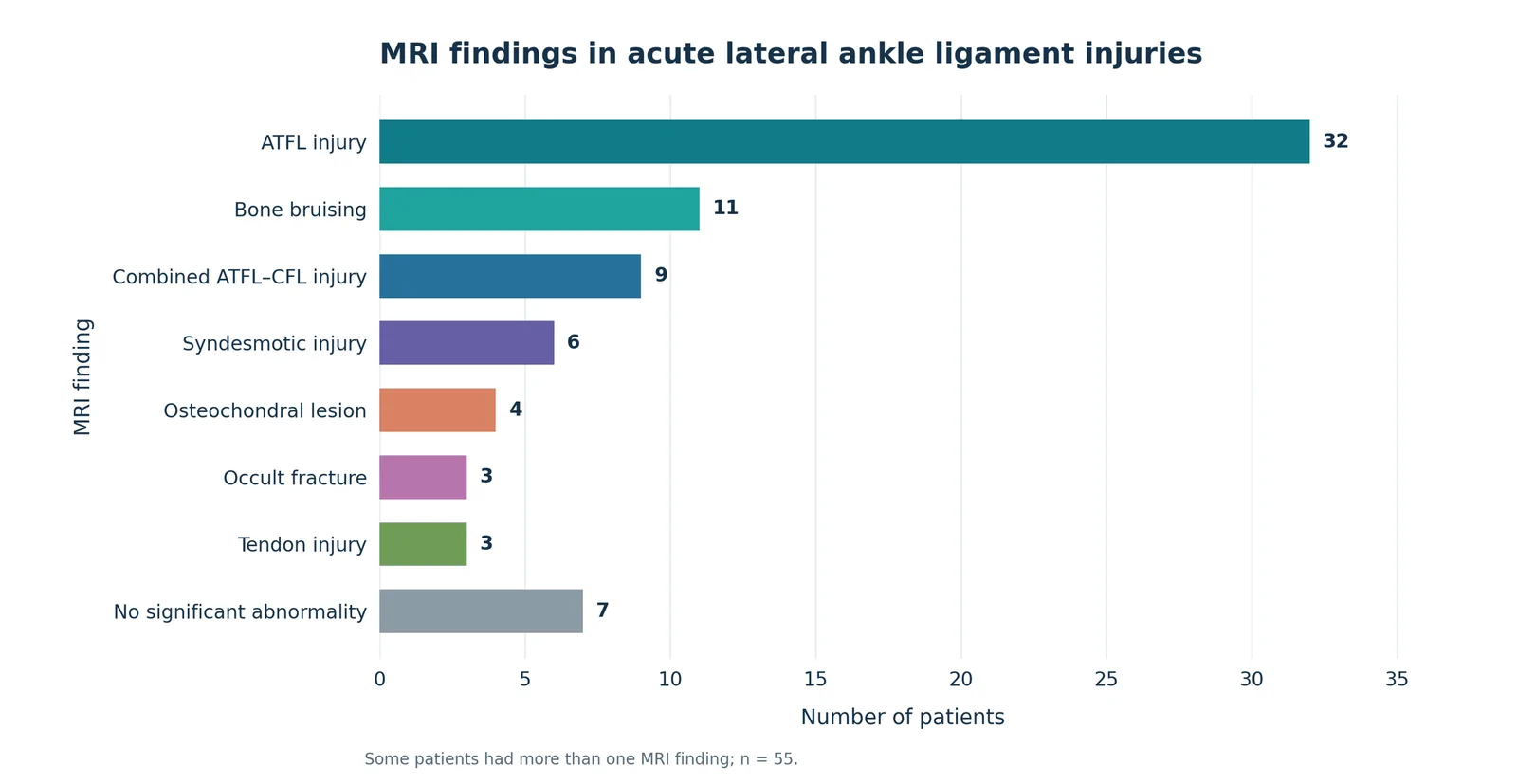
MRI findings in patients with acute lateral ankle ligament injuries (n = 55). The horizontal bar chart demonstrates the frequency of MRI findings in the study population. ATFL injury was the most frequently reported abnormality, occurring in 32 patients (58.2%). Because some patients had more than one MRI finding, the categories are not mutually exclusive, and the percentages do not total 100%. The graph was created using Microsoft Excel, version 16.113. Abbreviations: MRI, magnetic resonance imaging; ATFL, anterior talofibular ligament; CFL, calcaneofibular ligament.

**Figure 3:**
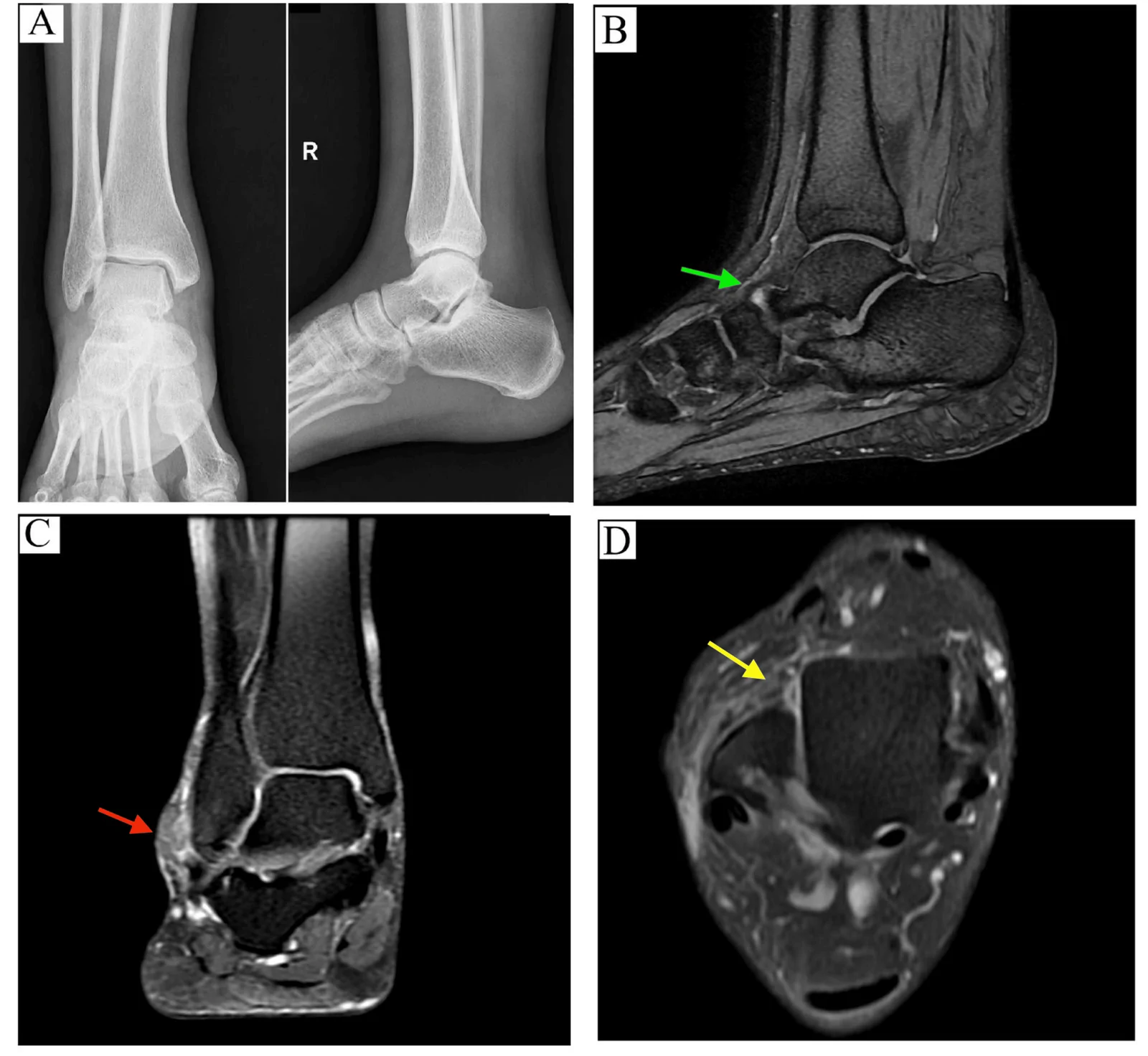
Representative radiographic and magnetic resonance imaging findings in a patient with acute lateral ankle ligament injury. (A) Anteroposterior and lateral radiographs of the right ankle showing preserved alignment without an acute fracture or dislocation. (B) Sagittal MRI showing increased signal and soft-tissue oedema in the anterolateral ankle region (green arrow). (C) Coronal MRI demonstrating oedema and abnormality within the lateral ligament complex (red arrow). (D) Axial MRI demonstrating abnormal signal and loss of the normal appearance of the anterior talofibular ligament, consistent with anterior talofibular ligament (ATFL) injury (yellow arrow). Source: De-identified radiographs and MRI from a patient included in this audit, obtained from the Kulhudhuffushi Regional Hospital imaging archive. MRI was requested because of persistent pain and swelling. Following MRI, management remained unchanged.

Correlation between clinical diagnosis and MRI

MRI confirmed the initial clinical diagnosis in 32 patients (58.2%). Additional clinically relevant findings not suspected during the initial assessment were identified in 13 patients (23.6%), while the primary diagnosis changed following MRI in six (10.9%). In the clinical diagnosis-MRI correlation classification, seven patients (12.7%) had no significant abnormality detected on MRI. The clinical diagnosis-MRI correlation categories were not mutually exclusive. Some patients had both confirmation of the initial diagnosis and additional clinically relevant findings. Consequently, category counts may exceed the total cohort size.

Initial management and impact of MRI

Before MRI, analgesics were prescribed to 51 patients (92.7%), ice, compression, and elevation were used in 39 (70.9%), functional ankle braces were provided to 34 (61.8%), and early mobilisation was initiated in 28 (50.9%). Walking boots were used in 14 patients (25.5%), while physiotherapy was initiated in 20 (36.4%). Patients could receive more than one treatment modality.

MRI findings resulted in a change in subsequent management in 19 patients (34.5%), while management remained unchanged in 36 (65.5%). Among patients whose management changed, targeted physiotherapy was initiated in eight, walking boot or prolonged immobilisation in seven, specialist follow-up was arranged in six, weight-bearing restrictions were introduced in four, surgical consultation was recommended in four, and additional CT imaging was requested in two. More than one management modification could occur in an individual patient.

Association between MRI appropriateness and management change

Among the 42 patients whose MRI requests met the predefined evidence-based criteria, management changed in 18 (42.9%), compared with one of 13 patients (7.7%) whose MRI requests were classified as potentially inappropriate based on the available documentation. MRI meeting the predefined evidence-based criteria was associated with significantly higher odds of a subsequent change in management (OR: 9.00, 95% CI: 1.07-75.70; Fisher's exact test, p = 0.022) (Table 5).

**Table 5:** Association between MRI appropriateness and subsequent change in patient management (n = 55). MRI meeting predefined evidence-based criteria was associated with higher odds of a subsequent change in patient management (OR: 9.00, 95% CI: 1.07-75.70; Fisher's exact test, p = 0.022). Abbreviations: MRI, magnetic resonance imaging; OR, odds ratio; CI, confidence interval.

MRI indication category	Management changed, n (%)	No change in management, n (%)	Total, n
Met predefined evidence-based criteria	18 (42.9)	24 (57.1)	42
Potentially inappropriate based on available documentation	1 (7.7)	12 (92.3)	13
Total	19 (34.5)	36 (65.5)	55

Overall audit compliance

Overall compliance is summarised in Table 6 and Figure 4. Three of seven standards met their predefined targets: plain radiography before MRI (55, 100%), mechanism of injury documented (53, 96.4%), and post-MRI management documented (53, 96.4%). Four targets were not met: weight-bearing status documented (43, 78.2%), ligament-specific examination documented (37, 67.3%), MRI meeting predefined evidence-based indications (42, 76.4%), and initial treatment documented (49, 89.1%).

**Table 6:** Overall compliance with predefined audit standards for the assessment and management of acute lateral ankle ligament injuries (n = 55). Observed performance was compared with the predefined target for each audit standard. Three of the seven standards met their predefined targets, while four did not. MRI examinations were considered to meet predefined evidence-based indications when at least one recognised indication was documented before imaging. Abbreviation: MRI, magnetic resonance imaging.

Audit standard	Target	Achieved	Compliance
Mechanism of injury documented	≥95%	53 (96.4%)	Met
Weight-bearing status documented	≥95%	43 (78.2%)	Not met
Ligament-specific examination documented	≥90%	37 (67.3%)	Not met
Plain radiography performed before MRI	100%	55 (100%)	Met
MRI met predefined evidence-based indications	≥90%	42 (76.4%)	Not met
Initial treatment documented	≥95%	49 (89.1%)	Not met
Post-MRI management documented	≥95%	53 (96.4%)	Met

**Figure 4:**
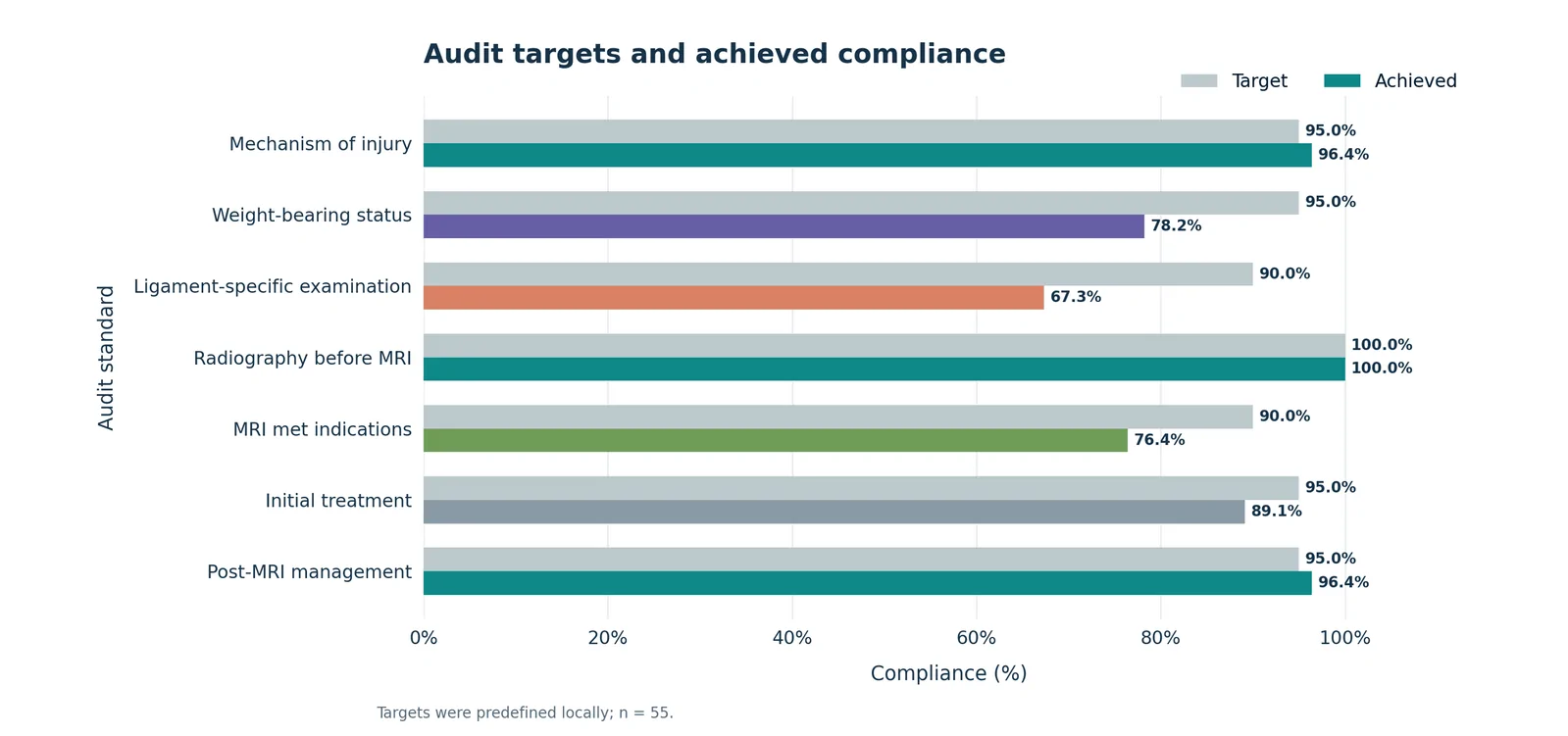
Comparison of predefined audit targets with achieved compliance for the assessment and management of acute lateral ankle ligament injuries. The figure compares achieved compliance with the predefined target for each audit standard among the 55 patients included in the clinical audit. Targets were achieved for documentation of the mechanism of injury (96.4% vs. ≥95%), plain radiography before MRI (100% vs. 100%), and documentation of the post-MRI management plan (96.4% vs. ≥95%). The remaining four standards did not meet their predefined targets. The graph was created using Microsoft Excel, version 16.113. Abbreviation: MRI, magnetic resonance imaging.

## Discussion

This clinical audit evaluated MRI utilisation in patients with acute lateral ankle ligament injuries and identified several opportunities to improve imaging selection and clinical documentation. All 55 patients underwent plain radiography before MRI, achieving complete compliance with the first-line imaging standard. However, only 42 MRI requests (76.4%) met the predefined evidence-based criteria, below the audit target of ≥90%. MRI altered subsequent management in 19 patients (34.5%), with management change occurring more frequently when MRI met evidence-based criteria than when no recognised indication was documented (42.9% versus 7.7%). MRI meeting predefined criteria was associated with higher odds of management change (OR: 9.00, 95% CI: 1.07-75.70; p = 0.022).

Complete compliance with plain radiography before MRI was consistent with current recommendations supporting radiography as the initial imaging modality when clinically indicated following acute ankle trauma [1,2,8,9,13]. MRI should generally complement rather than replace appropriate clinical and radiographic assessment. Nevertheless, approximately one-quarter of MRI examinations in this audit lacked a documented recognised indication. Because of the retrospective design, some patients may have had valid clinical indications that were not adequately recorded. These examinations are therefore more appropriately described as potentially inappropriate based on the available documentation rather than definitively unnecessary.

Clinical documentation represented an important area for improvement. Although the mechanism of injury was documented in 96.4% of patients, weight-bearing status was recorded in 78.2%, ligament-specific examination in 67.3%, syndesmotic testing in 40.0%, and the Ottawa Ankle Rules in only 29.1%. Current guidelines emphasise structured history, functional assessment, localisation of tenderness, and ligament-specific testing before advanced imaging [5-7]. A systematic review of clinical practice guidelines similarly supports structured assessment, functional treatment, exercise, and rehabilitation in the management of ankle sprains [14].

Syndesmotic injury warrants particular attention because diagnosis based on clinical examination alone can be challenging. Chang and Mandell described the complementary roles of radiography, CT, and MRI in assessing the distal tibiofibular syndesmosis [15]. In the present audit, syndesmotic testing was documented in only 40.0% of patients, whereas six patients (10.9%) subsequently demonstrated syndesmotic injury on MRI. Sman et al. reported that no single clinical test could reliably establish or exclude syndesmotic injury [16], while a subsequent meta-analysis by Netterström-Wedin and Bleakley similarly found that no individual test demonstrated both high sensitivity and high specificity [17]. These findings support a structured clinical assessment incorporating multiple examination findings before advanced imaging is considered.

The spectrum of MRI abnormalities in this audit was consistent with the expected pattern of acute lateral ankle injury. ATFL injury was the most frequent finding, occurring in 58.2% of patients, followed by bone bruising, combined ATFL-CFL injury, syndesmotic injury, osteochondral lesions, occult fractures, and tendon pathology. Existing systematic reviews support the diagnostic value of MRI for acute ATFL injury [10,12], while ultrasonography may provide an alternative ligament assessment modality in selected patients [11].

MRI may be particularly valuable when clinically significant associated pathology is suspected. Chun et al. reported greater sensitivity for MRI than radiography or CT for syndesmotic injury, with diagnostic performance approaching arthroscopic findings in the included studies [18]. Osteochondral lesions were identified in four patients (7.3%) in the present audit. Such lesions may present with persistent pain or mechanical symptoms after ankle trauma and may not be adequately demonstrated on initial radiographs; Haug et al. highlighted the role of MRI and other imaging modalities in their assessment [19]. These findings support selective MRI use when conventional assessment does not adequately resolve suspected syndesmotic, osteochondral, occult osseous, or other clinically important pathology.

An important finding was the association between MRI appropriateness and subsequent management change. Management was modified in 18 of 42 patients (42.9%) whose MRI fulfilled predefined indications compared with only one of 13 patients (7.7%) without a documented recognised indication. Changes included targeted physiotherapy, walking boot or prolonged immobilisation, weight-bearing restrictions, specialist follow-up, surgical consultation, and additional CT imaging. This suggests that careful patient selection may increase the likelihood that MRI contributes meaningfully to clinical decision-making rather than simply confirming an existing treatment plan.

The OR of 9.00 indicates a strong association between evidence-based MRI selection and management change; however, this finding should be interpreted cautiously. The wide 95% CI (1.07-75.70) reflects the small sample size, particularly the limited number of patients without a documented indication. Furthermore, the observational audit design cannot establish that appropriate MRI selection itself caused the management change. The finding therefore demonstrates an association rather than causation. A 2026 systematic review of 27 studies involving 1,931 ankles also reported high diagnostic accuracy of MRI for complete tears of the anterior and posterior inferior tibiofibular ligaments, while highlighting continuing uncertainty regarding imaging parameters for determining injury severity and prognosis [20].

Another area for improvement was initial rehabilitation. Physiotherapy was initiated before MRI in only 20 patients (36.4%). Contemporary clinical practice guidelines support early functional rehabilitation, progressive mobilisation, exercise therapy, and proprioceptive rehabilitation for most uncomplicated lateral ankle sprains [4,5,14]. More consistent implementation of structured rehabilitation may improve initial management and potentially reduce advanced imaging prompted primarily by persistent symptoms following incomplete conservative treatment.

The findings support several practical quality-improvement measures. A structured ankle assessment proforma should incorporate the mechanism of injury, weight-bearing ability, Ottawa Ankle Rules, localisation of tenderness, lateral ligament and syndesmotic examination, neurovascular assessment, and initial treatment. MRI requests should clearly document the specific clinical indication and diagnostic question. A standardised rehabilitation pathway, supported by clinician education on ankle examination, syndesmotic injury recognition, Ottawa Ankle Rule application, and evidence-based MRI indications, should also be implemented. Re-audit following these interventions will be necessary to determine whether clinical documentation and appropriate MRI utilisation improve and to complete the clinical audit cycle.

Limitations

This audit has several limitations. It was a retrospective, single-centre audit with a relatively small sample of 55 patients, limiting generalisability. Assessment of clinical practice depended on medical-record documentation; examinations may have been performed without being recorded, potentially underestimating compliance. In addition, only patients who ultimately underwent MRI were included in the final analysis, preventing assessment of MRI utilisation across the entire population presenting with acute ankle sprains. The audit evaluated short-term changes in management rather than long-term functional outcomes, recurrent instability, return to sport or work, patient-reported outcomes, or cost-effectiveness. Finally, MRI findings were derived from routine radiology reports, and interobserver agreement was not assessed.

Despite these limitations, the audit identifies modifiable deficiencies in clinical documentation and MRI selection and provides a practical framework for quality improvement. Re-audit after implementation of the proposed interventions will be important to assess improvement and complete the clinical audit cycle.

## Conclusions

This audit highlights the need for a clear clinical reason when requesting MRI after an acute lateral ankle injury. Documenting weight-bearing ability, ligament and syndesmotic examination findings, initial treatment, and the question that MRI is expected to answer can support more consistent imaging decisions and follow-up care.

A structured ankle assessment and MRI request pathway, supported by clinician education, may help address the gaps identified in this audit. Because the review relied on existing records, an undocumented indication may have been present in some cases. After the pathway is introduced, a re-audit should assess whether documentation and MRI selection improve.
